# Exploring the Bioactive Potential and Biocompatibility of Extracts from Agro-Industrial Residues for Cosmetic Applications

**DOI:** 10.3390/ijms26189169

**Published:** 2025-09-19

**Authors:** Sandra M. Gomes, Filipa Campos, M. Cristina L. Martins, Cláudia Monteiro, Lúcia Santos

**Affiliations:** 1 LEPABE—Laboratory for Process Engineering, Environment, Biotechnology and Energy, Faculty of Engineering, University of Porto, Rua Dr. Roberto Frias, 4200-465 Porto, Portugal; 2 ALiCE—Associate Laboratory in Chemical Engineering, Faculty of Engineering, University of Porto, Rua Dr. Roberto Frias, 4200-465 Porto, Portugal; 3 i3S—Instituto de Investigação e Inovação em Saúde, Universidade do Porto, Rua Alfredo Allen, 208, 4200-135 Porto, Portugal; 4 ICBAS—Instituto de Ciências Biomédicas Abel Salazar, Universidade do Porto, Rua de Jorge Viterbo Ferreira, 228, 4050-313 Porto, Portugal; 5 INEB—Instituto de Engenharia Biomédica, Universidade do Porto, Rua Alfredo Allen, 208, 4200-135 Porto, Portugal

**Keywords:** agro-industrial residues, antioxidants, biocompatibility, cosmetics

## Abstract

Every year, significant amounts of agro-industrial residues are generated. These residues contain several antioxidant compounds that can be extracted and applied to cosmetic products. In this study, phenolic-rich extracts from different agro-industrial residues (chestnut shell—CS, grape seed—GS, kiwi peel—KP, onion peel—OP, and pomegranate peel—PP) were obtained and their antioxidant potential and biocompatibility towards human fibroblasts (HFF-1) were evaluated. The total phenolic content ranged from 37.6 mg of gallic acid equivalents (GAE)/g for KP to 343.9 mg_GAE_/g for CS. Moreover, CS, GS, OP, and PP extracts exhibited strong antioxidant properties, while KP showed more moderate potential. Biocompatibility tests demonstrated that CS and GS extracts were non-cytotoxic at concentrations below 500 mg/L, while OP and PP were safe up to 1000 mg/L. KP extracts were biocompatible up to 10,000 mg/L. This work demonstrated the bioactive potential of various agro-industrial residues for application in the cosmetic industry, given their antioxidant capacity. Additionally, it was the first to establish safe application limits for Soxhlet-extracted compounds, ensuring their safety to consumers. This research emphasises the importance of evaluating the biocompatibility of each extract before its incorporation into cosmetics, as their composition is highly variable.

## 1. Introduction

In 2015, the United Nations established the 2030 Agenda for Sustainable Development, outlining a strategy to eradicate poverty and hunger while safeguarding the planet [1]. One of the goals of the Agenda is to guarantee sustainable consumption and production patterns (Sustainable Development Goal 12). This can be achieved by adopting Circular Economy principles, which involve reducing waste generation, namely agro-industrial residues, by repurposing and reusing them in various industries.

The agro-industrial sector generates around 1.3 billion tons of food waste per year, corresponding to one-third of the global food production [2]. Typically, this food waste is disposed of and incinerated in landfills, leading to the generation of toxic and greenhouse gases, negatively impacting the environment [3]. As a result, it is crucial to implement new sustainable approaches to mitigate these environmental impacts and to find ways to repurpose these residues for various applications. The agro-industrial residues, such as peels, seeds, and shells of fruits and vegetables, are usually rich in nutrients and bioactive compounds (BACs). These compounds offer various health benefits, including antioxidant and antibacterial [4]. In order to harness their potential biological benefits, agro-industrial residues can be incorporated into a wide range of products, such as food, cosmetics and pharmaceuticals [5,6] or used in the packaging of those products [7]. This approach supports the development of new sustainable products, which is aligned with the SDGs outlined by the United Nations.

Focusing on cosmetics, agro-industrial residues have the potential to be integrated into cosmetic formulations due to their biological properties, in particular their antioxidant capacity. The skin serves as a protective barrier, separating the body from the external environment. Skin ageing is an inevitable and complex biological process that results in the deterioration of the skin’s structural integrity and physiological functions, and can be induced by both internal and external factors. Internally, one of the primary causes of skin ageing is the accumulation of reactive oxygen species (ROS) in the electron transport chain of aerobic metabolism in the mitochondria of skin cells [6]. Antioxidants found in agro-industrial residues can counteract these molecules by transferring a hydrogen atom or an electron, which is possible given the resonance effects of the aromatic ring present in phenolic compounds (PC) [8]. The antioxidant potential of the BACs of agro-industrial residues can also be beneficial to the cosmetic formulations themselves. Cosmetics typically contain oils and fats, which are highly prone to lipid oxidation. This oxidation can negatively impact the physicochemical and sensory qualities of cosmetic products [9]. Therefore, incorporating ingredients with such properties may enhance the stability of the products.

Given the potential benefits of the BACs in agro-industrial residues to skin health and product stability, several authors have investigated their incorporation into cosmetic formulations. Table 1 presents examples of those studies.

The studies outlined in Table 1 demonstrate the benefits of incorporating phenolic-rich extracts into cosmetic formulations. However, when considering the incorporation of these extracts in products for human applications, it is vital to evaluate their biocompatibility to ensure consumer safety. Moreover, while there are regulations governing the type and amount of additives allowed in cosmetics [17], there are currently no regulations specifically addressing the addition of extracts from natural sources, such as agro-industrial residues, likely due to their variable composition. Although a few studies in the literature have assessed the biocompatibility of CS, GS, OP and PP extracts towards human fibroblasts [18,19,20,21], none of these studies used extracts obtained through Soxhlet extraction, as in the present work. Since the extraction method can impact the composition of the extracts and, therefore, their safety [22], this study aims to explore the potential of different Soxhlet-extracted compounds derived from agro-industrial residues in the cosmetic industry by exploring their antioxidant capacity and biocompatibility.

## 2. Results and Discussion

Phenolic compounds are well-known for their strong antioxidant properties [23]. Phenolic-rich extracts were obtained, using solid–liquid Soxhlet extraction, from five agro-industrial residues: chestnut shell (CS), grape seed (GS), kiwi peel (KP), onion peel (OP), and pomegranate peel (PP). Although 70–90% alcohols are most commonly used as extraction solvents in the literature, in this study 100% ethanol was chosen for two reasons: first, while ethanol can be evaporated using only the rotary evaporator, the addition of water requires an extra step of lyophilisation, which poses additional energetic and economic costs; second, mixing water and ethanol generates azeotropes, which makes it impossible to reuse the extraction solvent.

To assess the potential application of these extracts in the cosmetic industry, their total phenolic content (TPC) and antioxidant capacity (through their ability to inhibit the free radicals DPPH and ABTS) were evaluated. Three different batches of each extract were analysed and the results are presented in Table 2.

According to Table 2, it is possible to observe that CS was the extract with the highest TPC (344 mg_GAE_/g), followed by PP, OP and GS. KP presented a considerably lower TPC (38 mg_GAE_/g) than the other extracts. The TPC of an extract is greatly influenced by the extraction method and parameters (such as extraction time, sample-to-solvent ratio, and solvent) [24,25], as well as by the characteristics of the residue itself. Factors such as the soil and climacteric conditions under which the fruit/vegetable was grown, the ripening stage upon harvest, and the pre-treatment process (such as drying) can also impact the phenolic content [26]. As a result, a wide range of TPC values can be found in the literature for the extracts of the same agro-industrial residues evaluated in this work. For KP, the TPC is usually lower than 50 mg_GAE_/g [14,27,28]. For the other extracts, the values can vary from nearly 50 mg_GAE_/g to more than 800 mg_GAE_/g [29,30,31,32]. The values obtained in the present work are within the values found in the literature. These variations on the TPC may also explain the high standard deviation observed in the CS extract. In this study, three different batches of each extract were analysed, with four replicas for each batch. The high standard deviations derived from variations between the batches rather than variations among replicas of the same batch. Specifically, the results for the CS extract were as follows: batch 1: 344.0 ± 23.3 mg_GAE_/g; batch 2: 816.1 ± 20.7 mg_GAE_/g; and batch 3: 813.2 ± 32.9 mg_GAE_/g. These differences may arise because each extract is unique and variations in phenolic composition occur naturally in by-products due to, for example, exposure to different environmental factors, as explained above.

The TPC is related with the antioxidant potential of the extracts, with higher TPC corresponding to a greater antioxidant capacity. Here, the antioxidant properties of the extracts were analysed by measuring their ability to inhibit two free radicals (DPPH and ABTS), expressed as the extract concentration required to inhibit 50% of the respective radical (IC_50_) and also in comparison to a standard antioxidant, Trolox (mg_TE_/g). Once again, CS and PP demonstrated the best results (lower IC_50_ values and higher mg_TE_/g_extract_), followed by GS and OP, while KP showed the lowest antioxidant potency (higher IC_50_ value and lower mg_TE_/g_extract_). Despite the differences observed between extracts, all of them presented greater antioxidant capacity towards ABTS than DPPH and proved to be very strong antioxidants since the IC_50_ values were lower than 50 mg/L [33], with the exception of KP extract in the DPPH assay. Therefore, the extracts analysed are potential ingredients to enhance the antioxidant properties of cosmetic products. However, it is crucial to ensure the safety of these extracts before incorporating them into cosmetic formulations and applying them on human skin.

**Figure 1:**
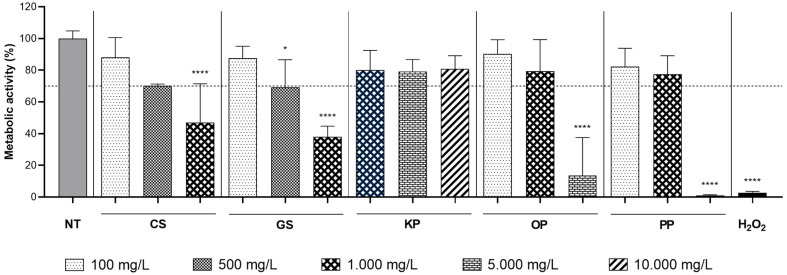
Cytocompatibility of phenolic extracts obtained from different by-products towards HFF-1 cells. NT—non-treated cells (positive control); cells incubated with extracts of chestnut shell (CS), grape seed (GS), kiwi peel (KP), onion peel (OP), and pomegranate peel (PP); H_2_O_2_—cells incubated with hydrogen peroxide (negative control). The results are expressed as mean ± standard deviation of three independent measures (*n* = 3). The dashed line represents 70% of metabolic activity according to ISO 10993-05. *, ****—represent significantly different values of metabolic activity compared to the non-treated cells (*p* < 0.05 and *p* < 0.0001, respectively).

The biocompatibility of the extracts towards human foreskin fibroblasts (HFF-1 cell line) was assessed and the results are illustrated in Figure 1.

The metabolic activity of HFF-1 cells was evaluated by resazurin assay after a 24 h-direct contact assay with different extracts (CS, GS, KP, OP, and PP) at different concentrations. Concentrations were selected based on the results of the antioxidant assays. The minimum concentration tested was set higher than the IC_50_ values obtained for each extract to ensure acceptable biological activity. Consequently, concentrations ranging from 100 to 10,000 mg/L were tested for CS, GS, OP, and PP, while concentrations from 1000 to 10,000 mg/L were tested for KP. According to ISO 10993-05, 70% of metabolic activity (in comparison to non-treated cells) is the threshold considered for cytocompatibility. As it is possible to observe in Figure 1, CS and GS extracts at concentrations up to 500 mg/L and OP and PP extracts up to 1000 mg/L did not induce any cytotoxic effect towards HFF-1 cell line. For KP, no cytotoxic effects were observed, even for the highest concentrations tested. Other studies have previously analysed the biocompatibility of these extracts towards human skin fibroblasts. For example, Lameirão et al. found that CS extracts were biocompatible at concentrations up to 1000 mg/L [18], while Monika et al. demonstrated that PP extracts caused cytotoxicity at 400 mg/L [21]. No literature reports were found regarding the cytotoxicity of KP extract on human fibroblasts. The differences between the literature and the results obtained in the present work could result from variations in the origin of the residues or differences in extraction conditions. In fact, no studies were found evaluating the biocompatibility of these extracts obtained using the Soxhlet extraction. This highlights the importance of evaluating the biocompatibility of each extract, as their composition is highly variable.

**Figure 2:**
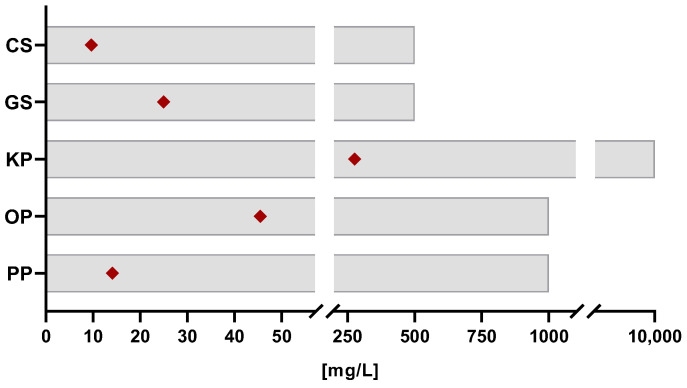
Correlation between the antioxidant capacity and the cytocompatibility of the extracts. The grey bars represent the range of concentrations where the extracts are safe towards human skin cells and the red dots represent the IC_50_ values of each extract regarding the DPPH assay.

Figure 2 correlates the results of the antioxidant capacity with the biocompatibility assay. It is possible to observe that, for all extracts, the IC_50_ values determined by the DPPH assay fall within safe concentration ranges. In fact, the concentration limit to prevent any cytotoxic effects is at least 20 times higher than the IC_50_ values. This ensures an extensive range of concentrations that can be used where the extracts present extremely powerful antioxidant properties within safe limits. Moreover, although the KP extract exhibited the lowest antioxidant potential (higher IC_50_ value), it had the widest range of non-cytotoxic concentrations for human fibroblasts. Therefore, higher amounts of KP extract can be incorporated into cosmetic products to achieve the same antioxidant effect as the other extracts without compromising the safety of consumers.

The findings of this study demonstrated the significant potential of different agro-industrial residues for application in cosmetic products. The strong antioxidant properties of the extracts could be beneficial in preserving cosmetic products and preventing human skin ageing and other oxidation-related skin problems. Additionally, this work identified non-cytotoxic concentrations for incorporating the extracts into cosmetic formulations while retaining their bioactive properties. Although the present study lays the groundwork for future research in this area, additional evaluations must be performed. In order to complement the fibroblast tests in 2D, additional biocompatibility assays should be conducted using more complex models (such as skin models, model organisms, and in vivo studies) to fully understand the effects of these extracts on the skin. Nevertheless, the cosmetics industry should consider these and other similar findings to maximise the antioxidant effects of its products, taking into account toxicity limits.

## 3. Materials and Methods

### 3.1. Samples and Reagents

Chestnut shells (CS) were provided by Agromontenegro company, located in Serra da Padrela (Alto Trás-os-Montes, Portugal). Grape seeds (GS) from different frape castes (Touriga Nacional, Touriga Francesa, and Tinta-roriz) were obtained from Alfândega da Fé, Bragança, Portugal. Kiwi peels (KP) were obtained from kiwis harvested in Viseu, Portugal (40°66′44″ N, 7°93′75″ W). Onion peels (OP) were obtained from onions harvested in Marco de Canaveses, Porto, Portugal (41°08′47″ N, 8°22′08″ W). Pomegranate peels (PP) were kindly provided by Honest Greens restaurant.

Gallic acid (Ref. 147915, C_7_H_6_O_5_, CAS 149-91-7), Folin–Ciocalteu reagent (Ref. 47641), potassium persulfate (Ref. 379824, K_2_S_2_O_8_, CAS 7727-21-1), (±)-6-hydroxy-2,5,7,8-tetramethylchromane-2-carboxylic acid (Trolox) (Ref. 238813, C_14_H_18_O_4_, CAS 53188-07-1), 2,2-diphenyl-1-picrylhydrazyl (DPPH) (Ref. D9132, C_18_H_12_N_5_O_6_, CAS 1898-66-4), and 2,2′-azino-bis(3-ethylbenzothiazoline-6-sulfonic acid) (ABTS) (Ref. A1888, C_18_H_24_N_6_O_6_S_4_, CAS 30931-67-0) were acquired from Sigma-Aldrich (St. Louis, MO, USA). Ethanol (Ref. 83813.360, C_2_H_6_O, CAS 64-17-5), acetic acid (Ref. 20104.312, C_2_H_4_O_2_, CAS 64-19-7) and sodium acetate (Ref. 27653.260, C_3_H_3_NaO_2_, CAS 127-09-3) were purchased from VWR (Radnor, PA, USA). Sodium carbonate (Ref. 13418, CNa_2_O_3_, CAS 497-19-8) was obtained from Honeywell (Charlotte, NC, USA). Foetal Bovine Serum (Ref. 10270106), Dulbecco’s Modified Eagle’s Medium (DMEM), trypsin, and Penicillin-Streptomycin (PenStrep) were purchased from Gibco (Cambridge, MA, USA).

### 3.2. Phenolic Compounds Extraction

A pre-treatment was applied to the samples prior to extraction. The agro-industrial residues (CS, GS, KP, OP, PP) were washed with running water and the excess water was removed with a paper towel. Afterwards, the samples were frozen at −80 °C for 24 h, following lyophilisation for 72 h. Finally, the samples were ground using a Q.5321 coffee grinder (Qilive, Auchan, Croix, France) to obtain a homogeneous powder with particles inferior to 1 mm. The obtained powder was used in the extraction process.

A Soxhlet extraction was performed for 2 h, according to the literature [14], to obtain a phenolic-rich extract. Ethanol was the solvent chosen and a sample-to-volume ratio of 1:20 m/V was applied. Solvent evaporation was achieved using a rotary evaporator (Rotavapor R-200, BUCHI Laboratories, Flawil, Switzerland), followed by a 2 mbar stream of nitrogen. The extracts were stored at 4 °C and protected from light using aluminium foil.

### 3.3. Extracts Characterisation

#### 3.3.1. Total Phenolic Content

The Folin–Ciocalteu assay was employed to determine the total phenolic content (TPC) of the extracts from different agro-industrial by-products, according to the literature [34]. The extract solutions (1000 mg/L) were mixed with the Folin–Ciocalteu reagent and left to incubate for 2 h at room temperature, after which the absorbance was measured at 750 nm using a spectrophotometer (UV-6300PC Double Beam Spectrophotometer, VWR, Radnor, PA, USA). A gallic acid calibration curve (50–1000 mg/L) was prepared to express the results in milligrams of gallic acid equivalents per gram of extract (mg_GAE_/g_extract_). The assay was performed in triplicate (three batches of each extract were analysed), each with four replicas.

#### 3.3.2. Antioxidant Activity

The antioxidant activity of the extracts was assessed through their ability to inhibit free radicals, specifically DPPH and ABTS, as described in the literature [34]. The ethanolic extracts solutions (CS: 1–200 mg/L, GS: 5–600 mg/L, KP: 200–5000 mg/L, OP: 5–1000 mg/L, PP: 1–600 mg/L, for DPPH; CS: 0.5–100 mg/L, GS: 1–200 mg/L, KP: 10–800 mg/L, OP: 1–200 mg/L, PP: 0.5–100 mg/L, for ABTS) were mixed with the radicals and left to incubate at room temperature (40 min for DPP; 15 min for ABTS). Finally, the absorbance was measured (515 nm for DPPH; 734 nm for ABTS), the inhibition percentage was calculated, and the results were expressed as the necessary concentration of extract to inhibit 50% of the free radical (IC_50_). The potential of the extracts to inhibit DPPH and ABTS radicals was compared to a standard antioxidant, Trolox (0.5–100 mg/L). The results were expressed as milligrams of Trolox equivalents per gram of extract (mg_TE_/g_extract_), by dividing the IC_50_ value of the Trolox by the IC_50_ value of the extract. The assay was performed in triplicate (three batches of each extract were analysed), each with four replicas.

### 3.4. Extracts Biocompatibility

The biocompatibility of the extracts towards human cells was assessed using the resazurin assay, according to the literature [35].

#### 3.4.1. Cell Culture

Complete Dulbecco’s modified Eagle’s medium (DMEM) with high glucose, stable glutamine and sodium pyruvate, supplemented with 10% (*v*/*v*) heat-inactivated foetal bovine serum and 1% (*v*/*v*) penicillin/streptomycin, was used to culture human foreskin fibroblasts (HFF-1, ATCC SCRC1041). The cells were maintained at a constant temperature of 37 °C and a CO_2_ level below 5%. Every two to three days, the culture medium was changed and, once the cells were nearly confluent, they were trypsinized, centrifuged for 5 min at 150× *g* and counted using a Neubauer chamber. Finally, the cells were seeded at a density of 13,000 cells/well in a 96-well tissue culture plate.

#### 3.4.2. Resazurin Assay

To evaluate the extracts’ cytotoxicity towards HFF-1 cells, a direct contact assay was conducted following the adapted ISO 10993-05 [36]. After 24 h of seeding, 20% of the culture medium was replaced by extract solutions of CS, GS, KP, OP, and PP at different concentrations (100–10,000 mg/L in ultrapure water for CS, GS, OP, and PP and 1000–10,000 mg/L for KP). After 24 h exposure at 37 °C, the supernatant was removed and resazurin 20% (*v*/*v*) was added to measure the metabolic activity of the cells. The plate was incubated at 37 °C, for 4 h, protected from light. After this time, 100 μL of each well were transferred to a 96-well black plate and the fluorescence was measured at an excitation/emission wavelength of 530 nm/590 nm, using a Synergy MX microplate reader (BioTek, Winooski, VT, USA). The culture medium was used as blank, cells treated with hydrogen peroxide (H_2_O_2_) served as negative control, and non-treated cells served as positive control. The assay was performed in triplicate (three batches of each extract), each with three replicates.

### 3.5. Statistical Analysis

GraphPad Prism 8.0.2 was used to perform the statistical analysis. Kruskal–Wallis test with Dunn’s multiple comparisons test was performed. Results were expressed as mean ± standard deviation and the values were considered statistically different when *p* < 0.05 at a 95% confidence interval.

## 4. Conclusions

The purpose of this work was to assess the potential of different agro-industrial residues (chestnut shell—CS, grape seed—GS, kiwi peel—KP, onion peel—OP, and pomegranate peel—PP) as ingredients for cosmetic formulations. Phenolic compounds can counteract skin ageing owing to their antioxidant properties and capacity to absorb UV radiation. Furthermore, these antioxidants can contribute to the stability of cosmetic formulations by preventing their degradation through oxidation. In this study, phenolic-rich extracts were obtained with different phenolic contents, ranging from 38 mg_GAE_/g in KP extracts to 344 mg_GAE_/g in CS extracts. The antioxidant properties of the five extracts were evaluated and CS, GS, OP, and PP extracts were found to be very potent antioxidants with IC_50_ values below 50 mg/L for both DPPH and ABTS assays. KP extract exhibited the lowest antioxidant potential, with IC_50_ values of 277 mg/L and 50 mg/L for DPPH and ABTS, respectively. Increasing extract concentrations were tested in contact with HFF-1 for 24 h and the impact on cell metabolic activity was assessed. The maximum extract concentrations with no observed cytotoxicity were determined: 500 mg/L for CS and GS, 1000 mg/L for OP and PP, and 10,000 mg/L for KP. These results are important to understand the concentration ranges within which each extract can be incorporated into cosmetic products, while maintaining exceptional antioxidant properties.

## Figures and Tables

**Table 1:** Studies on the incorporation of different extracts from agro-industrial residues into cosmetic formulations.

Agro-Industrial Residue	Objectives	Main Results	Ref.
Chestnut Shell (CS)	Evaluate the organoleptic and technological properties, as well as the stability, of an oil-in-water semisolid formulation containing chestnut shell extracts.	The formulation presented pleasant organoleptic properties, a skin-compatible pH, and suitable viscosity for topical application, with no impact on general stability.	[10]
	Assess the impact of a facial formulation containing chestnut shell extract on different human skin parameters.	The formulation improved the skin hydration, while slightly decreasing its roughness and wrinkles’ depth. The skin firmness increased after the application of the developed product.	[11]
Grape Seed (GS)	Study the use of grape seed extracts, in different concentrations (0.5, 1, 1.5, and 2% *w*/*w*), as an ingredient for cosmetic scrubs.	All formulations presented suitable spreadability and excellent physical stability. Scrubs enriched with grape seed extracts up to 1.5% did not cause skin irritation in vivo. The addition of the extract increased the total phenolic content and antioxidant activity of the scrubs.	[12]
	Evaluate the effectiveness of grape seed extract as a natural ingredient of sunscreen formulations for reducing skin age-related changes.	The application of the formulation containing the extract reduced melanin and erythema levels while improving skin tone, hydration and elasticity.	[13]
Kiwi Peel (KP)	Investigate the impact of incorporating kiwi peel extract into a moisturising cream.	The incorporation of the extract increased the antioxidant activity without affecting the product’s microbial safety and stability for two weeks.	[14]
Onion Peel (OP)	Examine the efficacy of onion peel extracts (free or encapsulated) as natural UV filters.	Onion peel extract and its microparticles conferred antioxidant properties to the sunscreen without affecting its stability. These natural ingredients proved to be effective in protecting the skin from UV radiation.	[15]
Pomegranate Peel (PP)	Assess the potential for creating a gel for topical application using pomegranate peel extract for wound healing and antimicrobial purposes.	Gels incorporated with pomegranate peel extracts presented antimicrobial properties against different microorganisms (*S. aureus*, *S. epidermidis*, *E. coli*, *C. albicans*). The formulation’s in vitro antimicrobial activity remained effective over 30 days against all tested strains.	[16]

**Table 2:** Antioxidant properties of phenolic extracts obtained from different by-products.

		CS	GS	KP	OP	PP
TPC (mg_GAE_/g_extract_)		343.9 ± 124.6 ^a^	208.5 ± 39.7 ^a^	37.6 ± 8.3 ^b^	243.8 ± 51.2 ^a^	260.3 ± 67.6 ^a^
DPPH	IC_50_ (mg/L)	9.6 ± 1.2 ^a^	25.0 ± 12.7 ^a,c^	276.8 ± 169.7 ^b^	45.5 ± 21.4 ^b,c^	14.1 ± 4.3 ^a^
	mg_TE_/g_extract_	413.5 ± 179.1 ^a^	169.0 ± 21.4 ^a,c^	16.2 ± 5.4 ^b^	93.9 ± 46.6 ^b,c^	270.6 ± 65.2 ^a^
ABTS	IC_50_ (mg/L)	3.5 ± 1.7 ^a^	6.5 ± 1.9 ^a,c^	49.9 ± 3.6 ^b^	10.0 ± 0.4 ^b,c^	4.3 ± 1.1 ^a^
	mg_TE_/g_extract_	657.8 ± 235.9 ^a^	324.8 ± 95.6 ^a,c^	39.1 ± 3.6 ^b^	194.6 ± 7.6 ^b,c^	478.3 ± 106.5 ^a^

CS—chestnut shell; GS—grape seed; KP—kiwi peel; OP—onion peel; PP—pomegranate peel; TPC—total phenolic content; GAE—gallic acid equivalents; DPPH—2,2-diphenyl-1-picrylhydrazyl; ABTS—2,2′-azino-bis(3-ethylbenzothiazoline-6-sulfonic acid); IC_50_—concentration of sample necessary to inhibit 50% of the radical; TE—Trolox equivalents. The results are expressed as mean ± standard deviation of three independent measures (*n* = 3). Different lowercase letters (a–c) represent statistically different values (*p* < 0.05) in the same line.

## Data Availability

The data supporting the conclusions of this article will be made available by the authors on request.
